# Extracorporeal Membrane Oxygenation for Secondary Organizing Pneumonia after Severe SARS-CoV-2 Infection: A Case Report

**DOI:** 10.3390/medicina57101013

**Published:** 2021-09-25

**Authors:** Tomoyuki Kimura, Chisato Onitsuka, Tomoko Kawahara, Yosuke Fukuda, Tetsuya Homma, Taro Watanabe, Koichi Ohsugi, Yuki Ichikawa, Atsuko Shono, Toru Kotani, Hironori Sagara

**Affiliations:** 1 Department of Medicine, Division of Respiratory Medicine and Allergology, Showa University School of Medicine, Tokyo 142-0064, Japan; psofriendtomo@hotmail.com (T.K.); chisato@med.showa-u.ac.jp (C.O.); coccy.ppt.1216@gmail.com (T.K.); terubow0423@gmail.com (Y.F.); sagarah@med.showa-u.ac.jp (H.S.); 2 Department of Intensive Care Medicine, Showa University School of Medicine, Tokyo 142-0064, Japan; watataro1978@gmail.com (T.W.); k.ohsugi@kpb.biglobe.ne.jp (K.O.); yukiichikawa8@gmail.com (Y.I.); atsukos@med.showa-u.ac.jp (A.S.); trkotani@med.showa-u.ac.jp (T.K.)

**Keywords:** SARS-CoV-2, COVID-19, organizing pneumonia, ECMO, dexamethasone

## Abstract

(Background) COVID-19 is caused by SARS-CoV-2 infection and may result in unfavorable outcomes. A recent large-scale study showed that treatment with dexamethasone leads to favorable outcomes in patients with severe COVID-19, and the use of extracorporeal membrane oxygenation (ECMO) has also been shown to improve outcomes. Recently, secondary organizing pneumonia (SOP) has been reported after SARS-CoV-2 infection, but the diagnostic and treatment strategies are still unclear. (Case presentation) Here, we report a patient with severe COVID-19 who developed SOP even after the use of dexamethasone, for whom the introduction of ECMO on the 19th day after hospitalization led to a favorable outcome. (Conclusions) Life-threatening SOP may evolve even after the use of dexamethasone, and the late-phase introduction of ECMO may save such patients with COVID-19.

## 1. Background

SARS-CoV-2-related pneumonia is a novel viral infection for which effective treatment strategies are urgently needed [1]. Extensive ground-glass opacity (GGO) accompanied by progressive hypoxemia is seen in severe cases and requires prolonged ventilatory management or extracorporeal membrane oxygenation (ECMO) [1].

Organizing pneumonia (OP) is a process of lung parenchymal injury of multiple etiologies, and it may be an idiopathic process known as cryptogenic OP. It usually occurs secondary to viral and bacterial infections, autoimmune diseases, and radiation therapy [2]. Recent studies reported that OP-like shadows were seen not only in the early phase of coronavirus disease 2019 (COVID-19), but also in the late phase. OP has been confirmed using both radiological and autopsy findings in patients with COVID-19 [3,4,5,6]. The first-line treatment for cryptogenic and secondary OP (SOP) is a systemic corticosteroid. However, the efficacy, safety, dosage, and length of therapy for COVID-19-related OP are still unclear [2,7], and guidelines for the late-phase introduction of ECMO among severe cases of OP are still to be determined.

A recent study showed that the use of dexamethasone at 6 mg/day for up to 10 days resulted in a lower 28-day mortality among hospitalized patients with COVID-19-related severe pneumonia on mechanical ventilation [7]. Although the use of systemic corticosteroids results in superior clinical outcomes, the mortality rate is still 29.3%, and additional therapy to improve outcomes is still debated [7]. In addition, the early introduction of ECMO for ARDS of any cause showed a mortality rate of 34%, but relative contraindications are mentioned in the Extracorporeal Life Support Organization (ELSO) guidelines when mechanical ventilation is used for more than 10 days [8,9].

Here, we present a case of worsening pneumonia, even with the use of dexamethasone and antiviral agents as the initial therapy, in which long-term systemic corticosteroid therapy and the introduction of ECMO showed favorable outcomes.

## 2. Case Presentation

**Figure 1:**
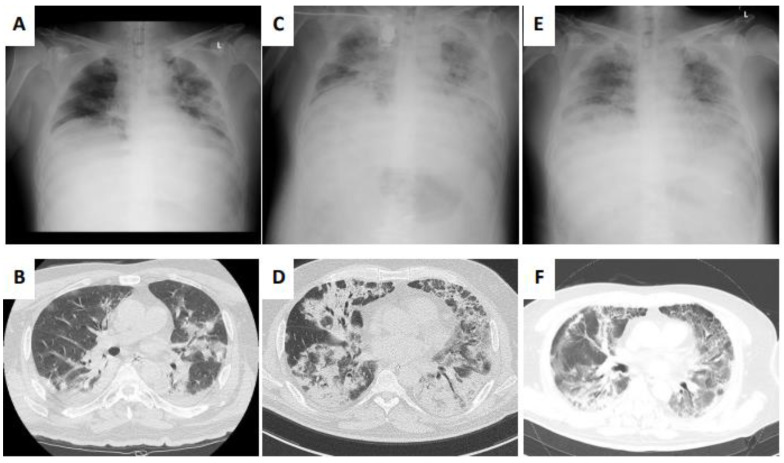
Chest images during the clinical course. (**A**) Chest X-ray and (**B**) computed tomography (CT) on admission. (**C**) Chest X-ray and (**D**) computed tomography (CT) at the time of SOP diagnosis. (**E**) Chest X-ray and (**F**) computed tomography (CT) on discharge.

A 56-year-old man, who was never vaccinated for SARS-CoV-2, with a medical history of hypertension treated with an oral calcium receptor antagonist, was admitted to an outside hospital with polymerase chain reaction (PCR) evidence of SARS-CoV-2 infection and had a fever. Treatment with favipiravir (initial dose of 3600 mg and 1800 mg/day from the second day) and dexamethasone (6.6 mg/day) was started without any use of anticoagulation. However, his respiratory condition worsened; therefore, he was transferred to our hospital the day after his initial admission. There was a progression to acute respiratory failure (PaO_2_/FiO_2_ (PF) ratio of 1.05), and the patient was placed on ventilator management (pressure-controlled ventilation with positive end expiratory pressure (PEEP) of 14 cm H_2_O and FiO_2_ of 1.0) and supine positioning on the day of admission (Figure 1A,B). In addition, remdesivir therapy (initial dose of 200 mg and 100 mg/day from the second day) was initiated, and dexamethasone was continued at 6.6 mg/day. With this treatment, oxygenation improved temporarily (PF ratio of 2.91) but worsened again on day 16 (PF ratio of 0.87), with progression of the GGO pattern on chest radiograph and elevation of the serum surfactant protein D (SP-D) from 116.4 to 804.7 ng/mL. Interestingly, other known OP-related biomarkers, such as Krebs von den Lungen-6 (KL-6) and lactate dehydrogenase (LDH), were not altered during the exacerbation of the respiratory condition. Chest computed tomography (CT) after the exacerbation showed extensive GGO with interlobular septal wall thickening and infiltrative shadows with peripheral bronchialectasis, which was compatible with SOP (Figure 1C,D). Although no significant bacteria were detected in sputum and bronchoalveolar lavage fluid (BAL) cultures, vancomycin (1250 mg/day) and meropenem (3 g/day) were added to the initial therapy for the risk of ventilator-associated pneumonia. SOP was diagnosed, veno-venous ECMO (V-V ECMO; jugulo-femoral) was introduced on the 19th day after admission, since a prone position did not alter the PF ratio, and a 14-day course of prednisolone (1 mg/kg, 70 mg/day) was begun with heparin (20,000 U/day). During the V-V ECMO run, pressure-controlled ventilation (initial PEEP and FiO_2_ were 12 cm H_2_O and 0.6, respectively) was kept. Prednisolone dosage was dropped from 70 mg per day to 10 mg per week and oxygenation improved. Finally, V-V ECMO was discontinued on the 19th day after its introduction (37th day after admission) and mechanical ventilation was discontinued on 20th day after decannulation of V-V ECMO (57th day after admission). The patient was discharged on the 121st day with long-term oxygen therapy (2 L/min) and a partial improvement was seen in chest CT findings (Figure 1E,F) and SP-D dropped to 258.1 ng/mL.

## 3. Discussion

Our patient developed OP even after sufficient systemic dexamethasone treatment, and the occurrence and progression of OP was diagnosed based on a chest CT, BAL, and serum SP-D. Long-term prednisolone therapy with ECMO support mitigated the deterioration in lung function, and the patient was discharged without severe pulmonary sequelae. The current case questions the dose and duration of initial systemic corticosteroid therapy, the optimal time to introduce ECMO, and the biomarkers used to diagnose severe COVID-19-related OP.

OP is a process of lung parenchymal injury that produces classic GGO findings on CT images [2]. Bacterial, fungal, parasitic, and viral agents, such as influenza, parainfluenza, and human immunodeficiency virus, can cause SOP [2]. The typical case of patients with OP displays a subacute disease course without a severe respiratory disorder [2,10]. Recent findings suggest that OP is seen in severe COVID-19, but the epidemiology and pathophysiology are still unclear. In addition, multiple studies have documented that CT findings are consistent with the OP pattern among patients with COVID-19 [4,5]. Fu et al. described that a second chest CT in 22 patients (71%) with COVID-19 showed improvement, while 4 patients had progression and 5 patients showed an unchanged picture [4]. Copin et al. studied postmortem lung specimens from six patients with COVID-19 and found that five patients showed a severe histological OP pattern, which was consistent with acute fibrinous and organizing pneumonia (AFOP) [5]. A systematic review of pathological findings among patients with COVID-19 showed that 59% of 131 cases had findings of AFOP [11]. The association between COVID-19 and OP, with possible progression to AFOP, is now becoming evident; however, the epidemiology is still unclear because pathological studies and autopsy cases are still scarce. These findings will certainly impact the outcome of COVID-19, and further study is warranted, since long-term systemic corticosteroid and ECMO support may alter the clinical course.

Corticosteroid therapy, which is the first-line treatment for both OP and COVID-19, improves symptoms, CT findings, and outcomes [2,12]. Recent findings from the RECOVERY trial supported the use of corticosteroids in patients with severe COVID-19, and other studies showed a decreased mortality and ventilator requirement [2,13]. Similarly, a meta-analysis showed that the use of hydrocortisone increased the odds of improvement in organ support-free days within 21 days and in the 28-day all-cause mortality among patients with severe COVID-19; however, it is still not clear whether this therapy is effective for SOP [14,15]. Further studies are warranted to clarify the need for long-term corticosteroid therapy for SOP due to COVID-19.

The World Health Organization (WHO) and ELSO recommended that ECMO should be considered for patients with COVID-19-related severe pneumonia or ARDS if lung-protective mechanical ventilation was insufficient to support oxygenation even with adequate drug administration [16,17,18,19,20,21,22,23]. An early introduction of ECMO among subjects with COVID-19 and ARDS is beneficial, but it is not clear whether the late-phase initiation of ECMO is beneficial for OP-related respiratory failure [24,25]. Compared with conventional ventilation, initiation of V-V ECMO reduced mortality (65% to 45%), such as young patients with fewer comorbidities, when started within seven days after intubation [26]. Although most contraindications are relative under normal circumstances, patients with a young age or failure of no more than one organ should be given priority for ECMO during a pandemic, when supplies and equipment are limited [27]. ECMO should be considered for SARS-CoV-2-induced SOP in the late phase of infection, since OP-related respiratory failure is usually reversible [28,29]. The introduction of V-V ECMO in patients with COVID-19-related SOP should be further studied, because systemic corticosteroid treatment might fail, as seen in our case.

Several serum biomarkers of OP severity have been reported, including KL-6, SP-A, SP-D, and LDH, but useful biomarkers have not been reported to detect SOP due to COVID-19 to date [27,30]. Yamagishi et al. showed that OP with high serum concentrations of SP-D relapsed more frequently than with low SP-D concentrations [27]. A recent case report by Horii et al. identified KL-6 and SP-D as possible biomarkers to evaluate SOP during COVID-19 treatment [28]. On the other hand, several prognostic biomarkers, including SP-D, have been identified among patients with COVID-19 [29,30]. Therefore, we speculate that patients with severe COVID-19, elevated SP-D levels, and CT evidence of OP will benefit from long-term prednisolone therapy; however, further clinical studies are warranted.

This case report has several limitations. It is based on a single case, and this exacerbation of pneumonia may have been due to the progression of initial infection, rather than SOP. Bronchoscopy may resolve this uncertainty by obtaining pathological tissue specimens, but the risk of viral infection should not be underestimated.

We believe that it is fair to suggest that the addition of corticosteroid therapy may alter the patient’s clinical course if SOP is suspected in the late phase of COVID-19, especially if severe hypoxemia is evident. Given this likely strong association between SOP or AFOP and COVID-19, systemic corticosteroid therapy and ECMO support should be carefully considered, since there are no reference standard therapeutic strategies for late-phase COVID-19-related infection. In clinical settings, OP or AFOP should be carefully diagnosed in patients with elevated serum SP-D concentrations and prolonged hypoxemia, because ECMO support may benefit such cases. Further research is warranted to clarify the diagnostic procedures, including biomarkers, and the treatment strategies for suspected SOP in patients with COVID-19.

## Data Availability

The data can be reached by contacting corrensponding author and the data is held at the institution.

## Abbreviations

GGO: ground-glass opacity; ECMO: extracorporeal membrane oxygenation; OP: organizing pneumonia; COVID-19: coronavirus disease 2019; SOP: secondary OP; ELSO: Extracorporeal Life Support Organization; PCR: polymerase chain reaction; PEEP: positive end expiratory pressure; PF: PaO2/FiO2; SP-D: surfactant protein D; KL-6: Krebs von den Lungen-6; LDH: lactate dehydrogenase; CT: chest computed tomography; BAL: bronchoalveolar lavage fluid; V-V ECMO: veno-venous ECMO; AFOP: acute fibrinous and organizing pneumonia; WHO: World Health Organization.
